# Assessing the antibiotic susceptibility of freshwater *Cyanobacteria* spp.

**DOI:** 10.3389/fmicb.2015.00799

**Published:** 2015-08-11

**Authors:** Elsa Dias, Micaela Oliveira, Daniela Jones-Dias, Vitor Vasconcelos, Eugénia Ferreira, Vera Manageiro, Manuela Caniça

**Affiliations:** ^1^National Reference Laboratory of Antimicrobial Resistances and Healthcare Associated Infections, Department of Infectious Diseases, National Institute of Health Dr. Ricardo JorgeLisbon, Portugal; ^2^Laboratory of Biology and Ecotoxicology, Department of Environmental Health, National Institute of Health Dr. Ricardo JorgeLisbon, Portugal; ^3^Centre for the Study of Animal Sciences, University of PortoPorto, Portugal; ^4^Laboratory of Ecotoxicology, Genomics and Evolution, Interdisciplinary Centre of Marine and Environmental Research, University of PortoPorto, Portugal; ^5^Faculty of Sciences, University of PortoPorto, Portugal

**Keywords:** cyanobacteria, antibiotics, minimum inhibitory concentrations, reduced susceptibility, water

## Abstract

Freshwater is a vehicle for the emergence and dissemination of antibiotic resistance. Cyanobacteria are ubiquitous in freshwater, where they are exposed to antibiotics and resistant organisms, but their role on water resistome was never evaluated. Data concerning the effects of antibiotics on cyanobacteria, obtained by distinct methodologies, is often contradictory. This emphasizes the importance of developing procedures to understand the trends of antibiotic susceptibility in cyanobacteria. In this study we aimed to evaluate the susceptibility of four cyanobacterial isolates from different genera (*Microcystis aeruginosa, Aphanizomenon gracile, Chrisosporum bergii, Planktothix agradhii*), and among them nine isolates from the same specie (*M. aeruginosa*) to distinct antibiotics (amoxicillin, ceftazidime, ceftriaxone, kanamycine, gentamicine, tetracycline, trimethoprim, nalidixic acid, norfloxacin). We used a method adapted from the bacteria standard broth microdilution. Cyanobacteria were exposed to serial dilution of each antibiotic (0.0015–1.6 mg/L) in Z8 medium (20 ± 1°C; 14/10 h L/D cycle; light intensity 16 ± 4 μEm^−2^s^−1^). Cell growth was followed overtime (OD_450*nm*_/microscopic examination) and the minimum inhibitory concentrations (MICs) were calculated for each antibiotic/isolate. We found that β-lactams exhibited the lower MICs, aminoglycosides, tetracycline and norfloxacine presented intermediate MICs; none of the isolates were susceptible to trimethoprim and nalidixic acid. The reduced susceptibility of all tested cyanobacteria to some antibiotics suggests that they might be naturally non-susceptible to these compounds, or that they might became non-susceptible due to antibiotic contamination pressure, or to the transfer of genes from resistant bacteria present in the environment.

## Introduction

Water environments are major pools of antibiotics/antibiotic resistant (AR) bacteria (Baquero et al., 2008) but the knowledge on the role of aquatic microorganisms on the dissemination/emergence of AR genes is still an under evaluated issue. Cyanobacteria are ubiquitous in aquatic ecosystems (Badger et al., 2006) and although they are exposed to antibiotic pollution/resistance (Martinez, 2009) it is not known if they may play a role on AR dissemination in natural ecosystems. Some reasons lead us to hypothesize that cyanobacteria may harbor AR genes: (1) they contain mobile genetic units such as transposable elements and plasmids (Chen et al., 2008; Christiansen et al., 2008; Lin et al., 2011), a requisite for horizontal gene transfer, the main mechanism of AR genes dissemination between distinct microbiota (Wright, 2007); (2) some cyanobacterial strains exhibit antibacterial activity (Madhumathi et al., 2011; Martins et al., 2011) and, as such, should have evolved mechanisms to protect themselves from their toxic action, i.e., they are “hot spots” for the evolution of AR (Wright, 2007); (3) some cyanobacterial strains were described as resistant to some antibiotics, including penicillin and amplicilin (Prasanna et al., 2010) and a penincilin-binding protein gene has been recently found in the cyanobacterium *Thermosynechococcus elongatus* (Urbach et al., 2008), though it remains to explain its weak β-lactamase activity and its physiologic role 4) it has been speculated that plasmids might determine cyanobacterial resistance to antibiotics (Chen et al., 2008), although this issue was never deeply evaluated.

The investigation of AR in bacteria usually integrates the evaluation of the susceptibility pattern to antibiotics and the search for resistance genotypes in those strains exhibiting a positive resistance phenotype. Several antimicrobial susceptibility testing approaches are standardized for the majority of bacterial pathogens (Mayrhofer et al., 2008), such as the Disk Diffusion (Matuschek et al., 2014) and Broth (Micro) dilution methods (ISO, 2006). Additionally, breakpoints or guidelines for interpreting the results from those tests are also harmonized among countries (CLSI, 2014; EUCAST, 2014) which facilitate the identification of resistant strains and the evaluation of emergence/dissemination of resistant bacterial pathogens worldwide.

Conversely, this is not the scenario for cyanobacteria since the issue of AR in cyanobacteria has been rarely investigated. The majority of studies regarding the effects of antibiotics on cyanobacteria are most related with the impact of antibiotic pollution upon aquatic ecosystems, some reporting harmful effects upon cyanobacteria (Halling-Sørensen et al., 2000; Pan et al., 2008; van der Grinten et al., 2010), but others reporting no deleterious effects (Stoichev et al., 2011). The relationship between antibiotics and cyanobacteria has also been addressed in the context of the establishment of axenic cultures. In fact, cyanobacterial cultures maintained in laboratory are usually non-axenic (Hong et al., 2010) since many water/environmental bacteria (ecosymbionts) are tightly attached to the mucilage of cyanobacteria colonies (Shiraim et al., 1989). Several methods have been proposed to obtain axenic cultures, generally involving the use of antibiotics or other chemical agents, UV radiation and physical separation approaches (Shiraim et al., 1989; Hong et al., 2010). However, the success of these purification procedures largely depends on the cyanobacteria isolates and their respective contaminants. On the other hand, testing cyanobacteria in solid media turns often a difficult task due to bacterial (over) growth since cyanobacteria exhibits lower growth rates than bacteria, within the range of 0.3–1.4 doubling per day (Mur et al., 1999). Additionally, cyanobacteria do not growth well on agar plates probably because agar might contain substances that inhibit cyanobacteria growth (Ferris and Hirsch, 1991; López-Rodas et al., 2006). Consequently, the methodologies standardized for susceptibility testing in bacteria are hardly applicable to cyanobacteria.

The information of cyanobacteria susceptibility to antibiotics is much dispersed and the available results cannot be easily compared given that very distinct experimental conditions and endpoints have been employed. In fact, cyanobacteria strains from very distinct species/habitats have been tested in distinct media, inocula, and temperatures; different antibiotic types and concentrations were used; a great variety of endpoints were employed to evaluate the effect of antibiotic on cyanobacterial growth such as chlorophyll/protein content; photosynthetic yield, cell number, optical density, among others. Besides, the results have also been expressed by different ways such as EC_50_, NOEC (no observed effect concentration) and resistant/susceptible phenotype (Reynaud and Franche, 1986; Liu et al., 2012; Kolar et al., 2014).

In this work we aimed to evaluate the susceptibility of several cyanobacteria species to antibiotics from distinct classes: β-lactams (amoxicillin, ceftazidime, ceftriaxone), aminoglycosides (gentamicin, kanamycin), quinolones (norfloxacin, nalidixic acid), tetracycline and trimethoprim, commonly used in the treatment of human/animal infectious diseases. We based our procedure on the conventional broth microdilution method used in antimicrobial susceptibility testing for bacteria (ISO, 2006), but optimized for the cyanobacteria culturing conditions. Data concerning the antibiotic residues in surface freshwaters is not well documented in Portugal, neither in other countries. However, the available information from environmental water samples revealed that antibiotics concentrations range from ng/L up to mg/L (Almeida et al., 2014), depending on the antibiotic and/or the sampling site (surface water, wastewater effluent, etc…). Among the above cited antibiotics we only found reported concentrations in Portugal for tetracycline (0.12–7 μg/L) (Novo et al., 2013; Varela et al., 2014), quinolones (0.03–4.4 μg/L) (Seifrtová et al., 2008; Novo et al., 2013) and trimethoprim (15.7 ng/L) (Madureira et al., 2010). On the other hand, few previous studies concerning the impact of antibiotics on cyanobacteria used the concentration range of mg/L (Vázquez-Martínez et al., 2004; Hong et al., 2010; Kolar et al., 2014). Based on these facts, we exposed the cyanobacteria to serial dilutions of the tested antibiotics within a concentration range of μg/L to mg/L, considering the worst scenario for the antibiotic contamination of freshwaters.

## Materials and methods

### Cyanobacterial strains

Strains of cyanobacteria, belonging to the “Estela Sousa e Silva Algae Culture Collection (ESSACC),” hereafter referred as LMECYA strains (Laboratory of Biology and Ecotoxicology, National Institute of Health, Portugal) were studied. LMECYA deposited in ESSACC have been characterized phylogenetically (Valério et al., 2009) and toxicologically (Pereira et al., 2001, 2004) and many have been used as reference strains in diverse studies (Paulino et al., 2009; Ballot et al., 2010; Martins et al., 2013).

The susceptibility to antibiotics was first evaluated in four strains from distinct genera. These strains were *Microcystis aeruginosa* (LMECYA 7), *Aphanizomenon gracile* (LMECYA 40), *Chrisosporum* (*Anabaena) bergii* (LMECYA 246), and *Planktothrix agardhii* (LMECYA 260), previously isolated from Portuguese freshwater resources with distinct geographical/utility characteristics: Montargil reservoir (rural area/recreational and agriculture activities), Crato reservoir (rural area/recreational and agriculture activities), Jamor lake (urban area/recreational activities), and São Domingos reservoir (rural area/agriculture and public supply), respectively. In a second step, the susceptibility to antibiotics was evaluated in eight additional strains of the same specie: the *M. aeruginosa*; these strains were isolated from rural freshwater reservoirs such as Montargil (LMECYA 91B, LMECYA 113, LMECYA 142), Roxo (LMECYA 50), Crato (LMECYA 108), Monte da Barca (LMECYA 151), Magos (LMECYA 159), and Corgas (LMECYA 167). All the isolates have been successfully maintained in the laboratory culture chamber as monoalgal, free of eukaryotes, non-axenic stock cultures, in Z8 medium (Skulberg and Skulberg, 1990), in a 14/10 h L/D cycle (light intensity 16 ± 4 μEm^−2^s^−1^, approx.) at 20 ± 1°C.

### Antibiotics preparation

In this study we tested the following classes of antibiotics: β-lactams (amoxicillin, ceftazidime, ceftriaxone), aminoglycosides (kanamycin, gentamicine), quinolones (nalidixic acid, norfloxacin), trimethoprim and tetracycline. Concentrated antibiotic stock solutions (25x–100x) were prepared by dissolving the powder antibiotics in sterile ddH_2_O, according to their solubility. These stock solutions were kept at −20°C. Antibiotic work solutions were prepared immediately before used and were diluted in ddH_2_O to the final concentration of 6.4 mg/L.

### Antibiotic susceptibility of four cyanobacteria genera

We used a methodology based on the standard microdilution method for bacteria (ISO, 2006), and adapted to the specific culturing conditions of cyanobacteria (LMECYA 7, LMECYA 40, LMECYA 246, and LMECYA 260), to evaluate their susceptibility to the antibiotics referred in Section Antibiotics Preparation. Briefly, we prepared a 96-well microplate for each antibiotic containing 100 μL of Z8 medium (Skulberg and Skulberg, 1990) in each well. Then, 100 μL of the antibiotic working solution (6.4 mg/L) was serial diluted (1/2) from column 1 to column 11 of the microplate (see Supplementary Figure 1). In parallel, 100 μL of Z8 medium were added to column 12 for the non-exposed cyanobacterial cells (control wells). Finally, 100 μL of each cyanobacterial isolate was inoculated in one microplate raw. We tested two inocula for each isolate corresponding to a final concentration of 5 × 10^5^ cells/mL (inoculum 1) and 2 × 10^6^ cells/mL (inoculum 2). Inoculum 1 corresponds to a biomass density within the range recommended by the OECD Guidelines for Testing of Chemicals in Freshwater Alga and Cyanobacteria (OECD, 2011). Considering that the effect of antibiotics might depend on cell densities, we also tested a four-time higher initial biomass (inoculum 2). These are cell densities commonly found in cyanobacterial blooms (Codd et al., 2005). The inocula were prepared by diluting cyanobacterial stock cultures in Z8 medium, according to their cell densities determined in Sedgewick-Rafter chambers by microscope counting (LeGresley and McDermott, 2010). Microplates were incubated in culture chamber at 20°C, 14/10 h light/dark cycle, light intensity 16 ± 4 μEm^−2^s^−1^.

Cyanobacterial cell density (OD_450*nm*_) (Churro et al., 2009) and microscopic examination of cultures integrity were followed for 13 days after incubation. Minimum inhibitory concentration (MIC) was considered as the lower antibiotic concentration that totally inhibited cyanobacteria cell growth (according to OD_450*nm*_ measures in relation to control wells and corresponding to the absence of undamaged cyanobacterial cells under microscopic examination). Few cyanobacterial colonies were still observed under microscope in some wells corresponding to putative MIC, although these wells exhibited negligible OD_450*nm*_ and no visible growth at naked eye. Consequently, we had to verify if those remaining cells were not viable in order to confirm the MIC value. For that purpose, 20 μL of all the microplate wells were re-inoculated in new microplates containing 180 μL of fresh Z8 medium per well and the cell growth was followed overtime (by OD_450*nm*_ measurement and microscopic examination) (confirmation step 1). We considered that the MIC value was maintained if no cyanobacterial growth was observed for that antibiotic concentration. If cell growth was still observed, we considered that the MIC value corresponded to the antibiotic concentration immediately above that inhibited cyanobacterial growth. Another growth control step was performed in order to confirm all the MIC values. For that purpose, the content of the wells corresponding to the putative MICs were re-inoculated in 4 well plates (dilution 1/2 in fresh Z8 medium) and the absence of cell growth was confirmed overtime (confirmation step 2). All the procedure was repeated in two independent experiments.

### Antibiotic susceptibility of eight *M. aeruginosa* strains

The same procedure (Section Antibiotic Susceptibility of Four Cyanobacteria Genera) was used to determine the MIC of antibiotics among the eight additional *M. aeruginosa* strains (referred at Section Cyanobacterial Strains), in order to evaluate antibiotic susceptibility trends within this specie. In this case, we tested the inoculum 1 in three independent experiments.

### Antibiotics quality control

To ensure that the antibiotics maintained their activity during the experiment course time, we performed a stability assay using the bacterial standard strains *Escherichia coli* (ATCC 25922) and *Staphylococcus aureus* (ATCC 29213), commonly used as quality controls of Antimicrobial Susceptibility Testing (EUCAST, 2014). For that purpose, we prepare 96-well microplates containing serial dilutions of the antibiotics stock solutions in Z8 medium within the respective MIC's range of those bacterial controls (Hakanen et al., 2002; ISO, 2006). These microplates were maintained in the cyanobacterial culture chamber conditions stated at Section Cyanobacterial Strains. Samples from those antibiotics were taken at 0, 1, 7, and 14 days and used to determine the MICs of *E. coli* and *S. aureus* strains by the standard Broth Microdilution procedure and interpreted according to the EUCAST guidelines (ISO, 2006; EUCAST, 2014).

## Results

### Antibiotic susceptibility of four cyanobacteria from different genera

#### Macroscopic observation of antibiotic-exposed cyanobacteria

The macroscopic observation of the cyanobacterial cultures exposed to the antibiotics (see Supplementary Figure 2) showed that their susceptibility depends on the type and concentration of the antibiotics. In a general way, the effect of the antibiotics on cyanobacterial growth followed three distinct patterns: strong decrease of cell viability at the lowest antibiotic concentrations (β-lactams, in particular amoxillicin), decrease of cell viability at middle/higher antibiotic concentrations (aminoglycosydes, tetracycline, and norfloxacine) and no effect at any antibiotic concentration (nalidixic acid and trimethoprim). Besides the type/concentration of the antibiotic, the effects were also dependent on the cyanobacterial strain with *M. aeruginosa* (LMECYA 7) being particularly susceptible to norfloxacin and *A. gracile* (LMECYA 40) and *C. berghii* (LMECYA 246) the most susceptible to β-lactams.

#### Dose-response curves

**Figure 1:**
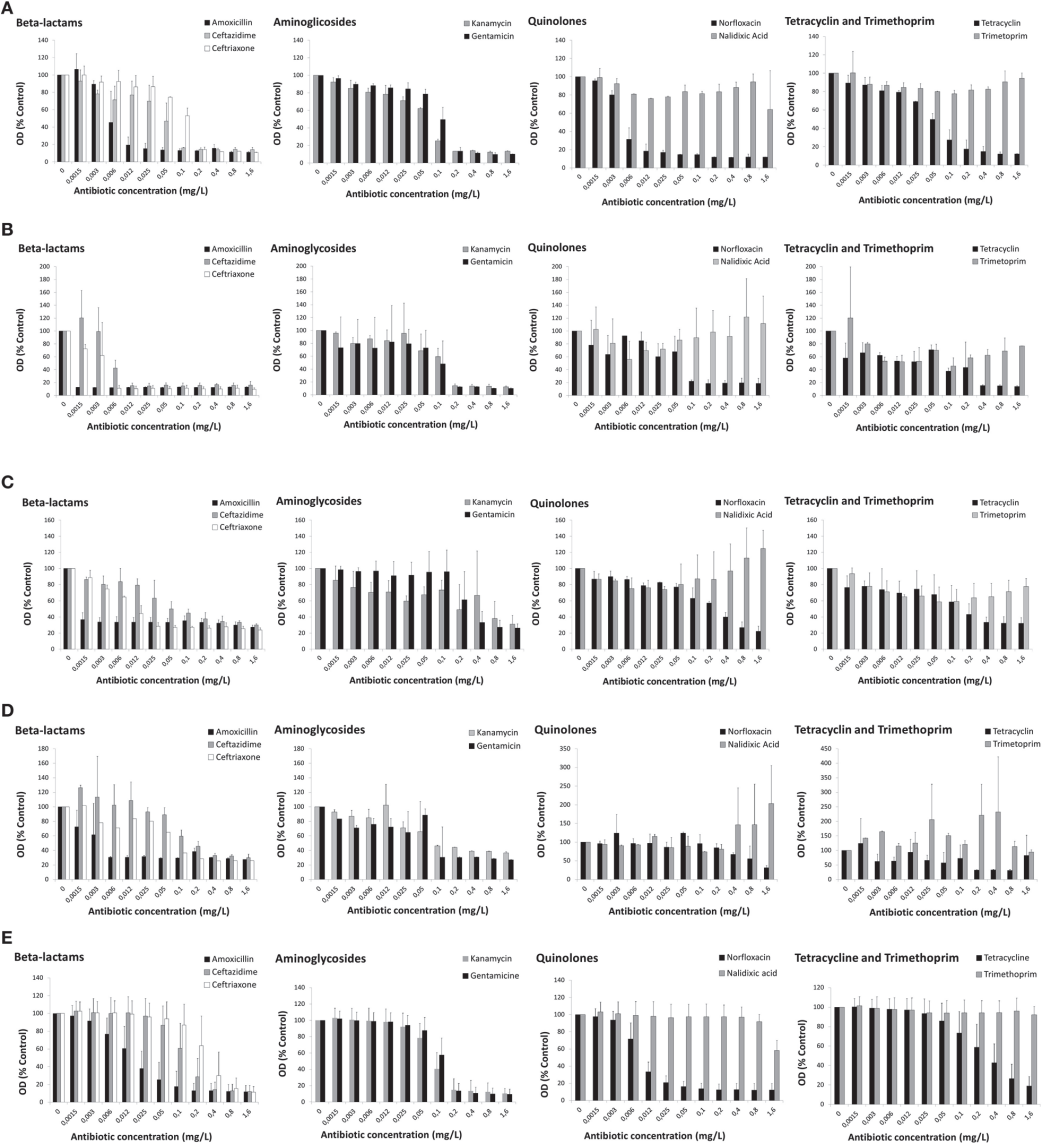
**Dose-response curves of cyanobacteria strains (5 × 10^5^cells/mL—inoculum 1) exposed to nine antibiotics from five distinct antibiotic classes for 13 days**. **(A)** *Microcystis aeruginosa* LMECYA 7; **(B)** *Aphanizomenon gracile* LMECYA 40; **(C)** *Chrisosporum berghii* LMECYA 246; **(D)** *Planktotjrix agardhii* LMECYA 260; **(E)** Mean values of nine *Microcystis aeruginosa* strains (LMECYA 7, 50, 91B, 108, 113, 142, 151, 159, and 167). In **(A–D)**, the data are expressed as values of optical density (450 nm) in relation to the control (non-treated cells) for each strain. Bars represent the mean plus standard deviation from two independent experiments, obtained for each antibiotic concentration (within the range of 0.0015–1.6 mg/L). In **(E)**, the data are expressed as the mean values of optical density (450 nm) in relation to the control (non-treated cells) of the nine LMECYA strains (7, 50, 91B, 108, 113, 142, 151, 159, and 167). Bars represent the mean plus standard deviation from three independent experiments (two for strain LMECYA7), obtained for each antibiotic concentration (within the range of 0.0015–1.6 mg/L).

The patterns of antibiotic susceptibility referred above (Section Macroscopic Observation of Antibiotic-exposed Cyanobacteria) were confirmed by the dose-response curves of each cyanobacterial strain after 13 days of exposure to the distinct antibiotic classes, as exemplified in Figures 1A–D for cultures corresponding to inoculum 1. The results are represented by the values of optical density (450 nm) expressed as the % of the control (non-treated cells).

The strain of *M. aeruginosa* (LMECYA 7) was particularly susceptible to amoxicillin and norfloxacin, exhibiting a strong reduction of cell density (approximately 80%) after exposure to 0.012 mg/L of these antibiotics (Figure 1A). Ceftazidime also induced a strong reduction of cell growth, but for 10-times higher antibiotic concentration, that is, 0.1 mg/L. Similar effects from ceftriaxone, aminoglycosides and tetracycline were produced at 0.2 mg/L.

The antibiotics belonging to the class of β-lactams induced a strong reduction on the growth of *A. gracile* (LMECYA 40), even at lowest tested concentration (0.0015-amoxicillin; 0.006 mg/L-ceftriaxone; 0.012 mg/L-ceftazidime). Norfloxacin (0.1 mg/L), aminoglycosides (0.2 mg/L), and tetracycline (0.4 mg/L) exerted similar effects, but for concentrations that are several orders of magnitude higher (Figure 1B).

The pronounced inhibition of *C. berghii* (LMECYA 246) growth was also observed at the lowest concentrations of amoxicillin (0.0015 mg/L) and ceftriaxone (0.025 mg/L). This strain was also susceptible to ceftazidime (0.05 mg/L), tetracycline (0.2 mg/L), aminoglycosides (0.4–0.8 mg/L), and norfloxacin (0.8 mg/L), but only after exposure to higher concentrations (Figure 1C).

The growth of *P. agardhii* (LMECYA 260) was severely affected by low concentrations of amoxicillin (0.006 mg/L), but the effect of the other two β-lactams, aminoglycosides and tetracycline was only observed at concentrations of 0.1–0.2 mg/L. Norfloxacin was effective against this strain at the higher concentration (1.6 mg/L) (Figure 1D).

As it can be depicted from Figure 1, none of the four cyanobacteria were susceptible to nalidixic acid and to trimethoprim in the tested concentrations (0.0015–1.6 mg/L).

The cyanobacteria cultures with higher inoculums (2 × 10^6^ cells/mL) exhibited similar dose-response patterns; but in some cases high concentrations of antibiotics were required to elicit the same effect on the reduction of cyanobacterial growth (data not shown). This aspect can be depicted from the MIC values, as presented below (Table 1).

**Table 1:** **Minimum inhibitory concentration (MIC mg/L) of distinct antibiotics on four cyanobacteria strains from ***Microcystis aeruginosa*** (LMECYA7), ***Aphanizomenon gracile*** (LMECYA 40), ***Chrisosporum bergii*** (LMECYA 246), and ***Planktothix agradhii*** (LMECYA 260)**.

**Minimum Inhibitory Concentration (MIC, mg/L)**																												
**Cyanobacteria**	**Inocula**	**Amoxicillin**			**Ceftazidime**			**Ceftriaxone**			**Norfloxacine**			**Nalidixic acid**			**Kanamycin**			**Gentamicine**			**Tetracyclin**			**Trimethoprim**		
		**ST^a^**	**CSt1^b^**	**CSt2^c^**	**ST^a^**	**CSt1^b^**	**CSt2^c^**	**ST^a^**	**CSt1^b^**	**CSt2^c^**	**ST^a^**	**CSt1^b^**	**CSt2^c^**	**ST^a^**	**CSt1^b^**	**CSt2^c^**	**ST^a^**	**CSt1^b^**	**CSt2^c^**	**ST^a^**	**CSt1^b^**	**CSt2^c^**	**ST^a^**	**CSt1^b^**	**CSt2^c^**	**ST^a^**	**CSt1^b^**	**CSt2^c^**
LMECYA 7	I1-A	0.025	0.05	0.8	0.1	0.1	0.4	0.2	0.2	>0.2	0.006	0.006	0.2	1.6	1.6	>1.6	0.1	0.1	0.2	0.2	0.2	0.4	0.8	0.4	>0.8	>1.6	>1.6	>1.6
	I1-B	0.006	0.025	>0.025	0.1	0.1	>0.2	0.2	0.2	0.2	0.012	0.012	0.012	>1.6	>1.6	>1.6	0.2	0.2	0.2	0.2	0.2	0.2	0.2	0.4	>0.4	>1.6	>1.6	>1.6
	I2-A	0.2	0.4	1.6	0.2	0.2	0.4	0.4	0.4	>0.4	0.012	0.012	0.2	>1.6	>1.6	>1.6	0.2	0.2	0.4	0.4	0.4	0.4	1.6	1.6	>1.6	>1.6	>1.6	>1.6
	I2-B	0.05	0.05	>0.2	0.1	0.1	0.2	0.4	0.4	0.4	0.012	0.012	0.025	>1.6	>1.6	>1.6	0.2	0.2	0.2	0.2	0.2	0.2	0.2	1.6	>1.6	>1.6	>1.6	>1.6
LMECYA 40	I1-A	0.0015	0.0015	0.003	0.012	0.006	0.012	0.006	0.006	>0.006	0.1	0.1	0.1	>1.6	1.6	>1.6	0.2	0.2	0.2	0.2	0.2	0.4	0.2	0.2	0.4	>1.6	>1.6	>1.6
	I1-B	0.0015	0.0015	0.003	0.006	0.012	0.012	0.006	0.006	0.006	0.1	0.1	0.1	>1.6	>1.6	>1.6	0.2	0.4	0.4	0.2	0.2	0.2	0.4	0.4	0.4	>1.6	>1.6	>1.6
	I2-A	0.003	0.003	0.006	0.012	0.012	>0.012	0.006	0.006	0.012	0.2	0.1	0.2	>1.6	>1.6	>1.6	0.4	0.4	0.4	0.4	0.4	0.4	0.8	0.8	0.8	>1.6	>1.6	>1.6
	I2-B	0.003	0.003	0.012	0.012	0.012	0.025	0.006	0.012	0.012	0.2	0.2	0.2	>1.6	>1.6	>1.6	0.4	0.4	0.4	0.2	0.2	0.2	1.6	1.6	1.6	>1.6	>1.6	>1.6
LMECYA 246	I1-A	0.0015	0.0015	0.003	0.2	0.025	0.05	0.025	0.025	0.05	0.8	0.2	0.8	>1.6	>1.6	>1.6	0.2	0.4	0.4	0.4	0.4	0.4	0.4	0.0015	0.8	>1.6	>1.6	>1.6
	I1-B	0.0015	0.0015	0.003	0.1	0.025	0.1	0.025	0.025	0.025	0.8	0.4	0.4	>1.6	>1.6	>1.6	1.6	0.8	1.6	0.4	0.4	0.4	0.4	0.2	0.4	>1.6	>1.6	>1.6
	I2-A	0.0015	0.0015	0.006	0.2	0.05	0.2	0.025	0.025	0.1	0.4	0.4	1.6	>1.6	>1.6	>1.6	0.4	0.4	0.8	0.2	0.2	0.4	0.8	0.8	>1.6	>1.6	>1.6	>1.6
	I2-B	0.006	0.006	0.012	0.4	0.4	0.4	0.05	0.05	0.05	1.6	0.8	0.8	>1.6	>1.6	>1.6	0.8	0.8	0.8	0.8	0.4	0.4	1.6	0.8	1.6	>1.6	1.6	>1.6
LMECYA 260	I1-A	0.003	0.003	0.003	0.4	0.1	0.2	0.05	0.1	0.1	0.8	0.8	1.6	>1.6	>1.6	>1.6	0.1	0.1	0.1	0.1	0.1	>0.1	0.05	0.05	0.1	>1.6	>1.6	>1.6
	I1-B	0.006	0.006	0.012	0.4	0.4	0.4	0.1	0.1	0.2	1.6	0.8	1.6	>1.6	>1.6	>1.6	0.1	0.2	0.2	0.1	0.1	0.1	0.2	0.2	0.4	>1.6	>1.6	>1.6
	I2-A	0.012	0.012	0.012	0.4	0.4	0.4	0.4	0.2	0.4	1.6	1.6	1.6	>1.6	>1.6	>1.6	0.2	0.1	0.2	0.1	0.2	0.8	0.2	0.2	>0.2	>1.6	>1.6	>1.6
	I2-B	0.012	0.025	0.1	0.4	0.4	0.8	0.2	0.2	0.4	0.8	0.8	1.6	>1.6	>1.6	>1.6	0.2	0.4	0.8	0.2	0.1	0.1	0.4	0.4	0.8	>1.6	>1.6	>1.6

a *St, Susceptibility Test*.

b *Cst1, Confirmation Step 1*.

c *Cst2, Confirmation Step 2*.

#### Minimum inhibitory concentrations (MIC)

Based on the dose-response curves obtained from the susceptibility test we determined the MIC of each antibiotic for each cyanobacterial strain, as shown in Table 1. In these table we indicate the MIC value obtained for inoculum 1 and 2 (I1 and I2, respectively) of the four cyanobacterial isolates (LMECYA 7, LMECYA 40, LMECYA 246, and LMECYA 260) after performing two independent experiments (A and B). Besides, and as explained in Section Antibiotic Susceptibility of Four Cyanobacteria Genera, we had to confirm those MIC values by the confirmation step 1 and confirmation step 2, whose results are also included in the tables.

The MIC values obtained with the microplate susceptibility assay agree, in a general way, with those obtained after performing the two additional confirmation steps. Punctually, however, the MICs obtained by confirmation steps 1 and 2 were higher. That is the case of LMECYA 7 exposed to amoxicillin and norfloxacin. We attribute these discrepancies to the globular shape of the LMECYA 7 colonies. It is known that colony formation in *Microcystis* spp. relates to species survival, with the outer cells protecting the colony from photo-inhibition and predators but being the inner cells potentially limited in terms of light and nutrients (Mulling et al., 2014). We hypothesize that amoxicillin and norfloxacin might not have reached the inner cells of the colonies with higher dimensions, which enabled those remaining living cells to growth after re-inoculation in fresh Z8 medium during the confirmation steps procedures. We suppose that this problem did not occur with LMECYA 40, LMECYA 246, and LMECYA 260 since this species form filamentous colonies, being all cells equally exposed to the surrounding medium. Since the MIC corresponds to the antibiotic concentration that inhibits completely the cell growth, we considered that the MIC values of our experiments were those obtained by the confirmation step 2.

As depicted from Table 1, the cyanobacteria LMECYA 40, LMECYA 246, and LMECYA 260 were particularly susceptible to the lowest concentrations of amoxicillin, exhibiting MIC values between 0.003 and 0.1 mg/L. The cyanobacteria LMECYA 7 was the less susceptible to this antibiotic (0.025 < MIC ≤ 1.6 mg/L). The MIC values of ceftriaxone were also low for LMECYA 40 (0.006–0.012 mg/L) and LMECYA 246 (0.025–0.1 mg/L). Ceftazidime was particular effective against LMECYA 40 (0.012–0.025 mg/L). In conclusion, the strain LMECYA 40 was the most susceptible cyanobacteria to the β-lactam antibiotics, whereas LMECYA 7 was the less susceptible.

The MIC values for aminoglycosides were, in a general way, higher than those from the β-lactams and did not differ considerable between cyanobacteria strains. The MIC of aminoglycosides varied between 0.1 and 0.8 mg/L (LMECYA 260), 0.2–0.4 mg/L (LMECYA 7 and LMECYA 40), and 0.4–1.6 mg/L (LMECYA 246). The same was found for tetracycline, with MIC values between 0.1and 0.8 mg/L (LMECYA 260) and 0.4–1.6 mg/L (LMECYA 40 and LMECYA 246). For LMECYA 7 the MIC of tetracycline was higher than 1.6 mg/L.

Norfloxacin was particularly effective in the inhibition of LMECYA 7 growth (0.012 ≤ MIC ≤ 0.2 mg/L) and less effective against LMECYA 260 (*MIC* = 1.6 mg/L).

Within the concentration range of the tested antibiotics (0.0015–1.6 mg/L) it was not possible to determine a MIC value for nalidixic acid and trimethoprim, since cyanobacterial growth was consistently observed for all the four tested cyanobacteria.

### Comparative study of the antibiotic susceptibility of nine *M. aeruginosa* strains

#### Dose-response curves

The effect of the several antibiotics on the growth of the *M. aeruginosa* strains is represented in Figure 1E. The results, expressed as the mean optical density (% control) of the nine tested LMECYA strains (7, 50, 91B, 108, 113, 142, 151, 159, and 167), were quite consistent among *M. aeruginosa* strains. These results also show that the effect of antibiotics followed a similar pattern of that obtained previously with LMECYA7 (Figure 1A). Among β-lactams, all strains were more susceptible to amoxicillin and less susceptible to ceftriaxone. As for LMECYA7, all the other *M. aeruginosa* strains were particularly susceptible to low concentrations of norfloxacin (0.025 mg/L). Aminoglycosides induced a strong reduction of cyanobacterial growth at 0.2 mg/L. These strains exhibited low susceptibility to tetracycline and their growth was not affected by nalidixic acid and trimethoprim.

#### Minimum inhibitory concentrations

The MICs of the nine antibiotics tested against *M. aeruginosa* strains are shown in Table 2. The lower MIC of norfloxacine was 0.05 mg/L for four of the nine strains and the concentration of 0.2 mg/L inhibited all the strains. The MICs of kanamycine, ceftazidime, and amoxicillin were within the following ranges, respectively: 0.1–0.4, 0.1–0.8, and 0.1–1.6 mg/L, where the antibiotics at concentrations of 0.4, 0.8, and 1.6 inhibited 100% of the strains. The lowest concentration of gentamicine and ceftriaxone that inhibited *M. aeruginosa* was 0.2 mg/L and the highest (with 100% of inhibition) was 0.4 and 1.6 mg/L, respectively. These susceptibility results have the same tendency as those from LMECYA 7 strain (Table 1). Similarly, none of the strains were inhibited by nalidixic acid, tetracycline and trimethoprim in a concentration up to 1.6 mg/L.

**Table 2:** **Minimum inhibitory concentrations of the nine strains of ***M. aeruginosa*****.

***M. aeruginosa* starins**	**MIC (mg/L)^a^^,^^b^**								
	**AMX**	**CAZ**	**CRO**	**KAN**	**GEN**	**NOR**	**NAL**	**TET**	**TMP**
LMECYA 7	0.8	0.4	0.2	0.2	0.2	0.05	>1.6	>0.8	>1.6
LMECYA 50	0.2	0.8	0.8	0.2	0.2	0.2	>1.6	>1.6	>1.6
LMECYA 91B	0.1	0.1	1.6	0.2	0.2	0.05	>1.6	>1.6	>1.6
LMECYA 108	0.2	0.8	0.8	0.1	0.2	0.1	>1.6	>1.6	>1.6
LMECYA 113	0.1	0.4	0.8	0.4	0.4	0.05	>1.6	>1.6	>1.6
LMECYA 142	0.2	0.2	0.8	0.2	0.2	0.2	>1.6	>1.6	>1.6
LMECYA 151	1.6	0.2	0.8	0.2	0.2	0.1	>1.6	>1.6	>1.6
LMECYA 159	0.1	0.2	0.4	0.2	0.2	0.05	>1.6	>1.6	>1.6
LMECYA 167	0.2	0.8	0.8	0.2	0.2	0.2	>1.6	>1.6	>1.6

a *Median values of the three independent experiments*.

b *AMX, amoxicillin; CAZ, ceftazidime; CRO, ceftriaxone; KAN, kanamycin; GEN, gentamicine; NOR, norfloxacine; NAL, nalidixic acid; TET, tetracycline; TMP, trimethoprim*.

## Quality control of antibiotic activity

The activity of the majority of the tested antibiotics was not influenced by the culture conditions of cyanobacteria during the time course of the susceptibility assay. In fact, and according to ISO guidelines (ISO, 2006), the MICs for these antibiotics were those expected for at least one of the standard bacterial strains (*E. coli* ATCC 25922 and *S. aureus* ATCC 29213) (see Supplementary Table 1). This ensures that the weak response and/or the unresponsiveness of cyanobacteria for those antibiotics were not due to the loss of antibiotic activity or antibiotics degradation. The exceptions were tetracyclin and amoxicillin, which MICs were not maintained after 7 and 14 days in both ATCC strains. Nevertheless, cyanobacteria were sensitive to these two antibiotics, reflecting their irreversible effect produced, at least, during the first 24 h of exposure, while their potency was maintained.

## Discussion

Cyanobacteria are primary producers in freshwater ecosystems, playing important roles such as oxygen production, nitrogen fixation and nutrients supply (Badger et al., 2006; González-Pleiter et al., 2013). Considering that cyanobacteria might be particularly vulnerable to water contaminants (López-Rodas et al., 2006; González-Pleiter et al., 2013) it becomes crucial to evaluate how these organisms, and consequently the aquatic ecosystems, are impacted by antibiotic pollution. In fact, there have been an increasing concern with the ecological risk of antibiotics in the aquatic environment (van der Grinten et al., 2010) and cyanobacteria have been recommended as a test organism to predict the concentration of antibiotics in the environment for which adverse effects are not expected to occur (EMEA, 2006). This recommendation was based on the assumption that cyanobacteria are sensitive to antibiotics. However, the exposure of cyanobacteria to antibiotics has never been approached from the standpoint of resistance to antibiotics, at least in a systematic way, and the role of cyanobacteria on water resistome was never evaluated.

In this work, we observed that the response of freshwater cyanobacteria to antibiotics depends on the type/concentration of the antibiotic as well as on cyanobacteria isolate, using conditions corresponding to a realistic scenario of bloom formation and of antibiotic contamination levels in freshwater.

Our results demonstrated that the β-lactams were the most effective antibiotics against three of the four tested cyanobacteria genera, exhibiting the low MIC values (from 0.003 to 1.6 mg/L). This agrees with previous studies pointing out deleterious effects of β-lactams on cyanobacterial strains. For example, the 50% effective concentration (EC_50_) of amoxicillin in *M. aeruginosa* growth rate after 7 days of exposure was reported as 0.0037 mg/L (Lützhøft et al., 1999) and 0.008 mg/L (Liu et al., 2012). Although these values were obtained by different endpoints of toxicity (cell density and chlorophyll, respectively), they falls within the range of the MICs determined in our study. Additionally, it was described that cyanobacterial cell wall can be disrupted by the enzymatic actions of penicillin (Holm-Hansen, 1968) and that amoxicillin can impair photosynthesis in cyanobacteria (Pan et al., 2008). Conversely, it was found that the marine *Phormidium valderianum* is able to use ampicillin as a nitrogen source, which confer it a resistance phenotype to this antibiotic up to 2 mg/mL (Prabaharan et al., 1994). The resistance to ampicillin, carbenicillin, and penicillin (10 mg/L) was also reported for *Gloeocapsa* sp. and *Chroococcidiopsis* sp. (Reynaud and Franche, 1986). Besides, the ability to produce β-lactamases was hypothesized for *Lyngbya spiralis, Anabaena variabilis* and *Calothrix membranacea* and *T. elongatus* (Urbach et al., 2008; Padmapriya and Anand, 2010). A screening with *Anabaena* sp. and *Nostoc* sp. using the agar double layer method showed that both susceptible and resistant patterns were observed for some β-lactams (30 μg), depending on the strain and/or the antibiotic (Prasanna et al., 2010). The available information is still somehow contradictory and the effects of β-lactams on cyanobacteria are far for being elucidated.

According to our results, aminoglycosides exhibited MICs ranging from 0.1 to 1.6 mg/L with no apparent differences between cyanobacteria. These results agree partially with previous data. In fact, Cameron and Pakrasi (2011) showed that *Synechocystis* sp. is susceptible to gentamicine (1–10 mg/L). However, a resistance phenotype was shown for *Synechococcus* sp. exposed to 10 mg/L (Reynaud and Franche, 1986). Similarly, susceptibility to 30 μg of kanamycine was reported for *Anabaena* sp., *Nostoc* sp., *Synechochoccus* sp. and *Pseudoanabaenacea* (Lorenz and Krumbein, 1984; Prasanna et al., 2010), but studies related with the axenic purification of marine cyanobacteria have shown that the growth of *Nodularia* sp. (Hong et al., 2010) and *Phormidium animalis* (Vázquez-Martínez et al., 2004) was maintained after exposure 100 and 150 mg/L of kanamycine, respectively. It is known that, besides their principal mechanism of action (inhibition of protein synthesis), aminoglycosides can also cause cytotoxicity through the induction of reactive oxygen species (Cameron and Pakrasi, 2011). Recently, it was suggested that glutathione contributes to gentamicine resistance in the cyanobacterium *Synechocystis* sp. (Cameron and Pakrasi, 2011). In fact, cyanobacteria encode specific glutathione transferases and have high levels of citosolic gluthathione (Wiktelius and Stenberg, 2007). However, if glutathione may underlie the resistance of few cyanobacteria to aminoglycosides, it does not explain the susceptibility of other strains.

A review of the literature also shows that different degrees of susceptibility to tetracyclines were reported for some cyanobacteria. *Anabaena* spp., *Nostoc* sp., and *Synechochoccus* sp. were described as susceptible to 30–50 μg/disk of tetracycline (Lorenz and Krumbein, 1984; Prasanna et al., 2010). According to toxicity tests performed by OECD guidelines (OECD, 2011), an EC_50(72*h*)_ value of 2.7 mg/L for oxitetracycline was calculated for *Anabaena flos-aquae* (Kolar et al., 2014) and an EC_50(7*days*)_ of 0.09 mg/L for tetracycline was found in *M. aeruginosa* (Halling-Sørensen, 2000). On the other hand, a study showed that the photosynthetic efficiency *M. aeruginosa* was not affected by oxytetracycline in the range of 0.001–1 mg/L (van der Grinten et al., 2010). Some of this data fall within the MIC range for tetracycline obtained with our LMECYA isolates (some within the range of 0.1–1.6 mg/L; others with MIC > 1.6 mg/L).

Our results led us to hypothesize that the cyanobacteria might be intrinsically non-susceptible to trimethoprim and nalidixic acid (MIC > 1.6 mg/L). Trimethoprim exerts its antibiotic activity by inhibiting the dihydrofolate reductase (folA), an essential enzyme involved in folate metabolism in prokaryotes (Myllykallio et al., 2003). However, it was previously demonstrated that several bacteria and two strains of cyanobacteria (*Nostoc* sp. PCC7120 and *Synechocystis* sp. PCC6803) lacks genes encoding *folA* and that they have an alternative pathway (thymidylate synthase, ThyX) to synthesize reduced folate molecules required for intermediary metabolism (Myllykallio et al., 2003). Inclusive, strains of *Helicobacter* spp. and *Campylobacter* spp. lacking *folA* and using ThyX for nucleotide synthesis were considered endogenously resistant to low levels of trimethoprim (Myllykallio et al., 2003). If the presence of alternative pathways to *folA* is a common feature of cyanobacteria, then we can postulate that cyanobacteria are naturally non-susceptible to trimethoprim. Actually, previous studies have shown that some cyanobacteria exhibit reduced susceptibility to this antibiotic. A microplate test with *M. aeruginosa* revealed that cyanobacterial growth is not inhibited after 24 h of exposure to trimethoprim within the range of 0.001–10 mg/L (*EC*_50_ = 6.9 mg/L) using the photosynthetic yield as endpoint of antibiotic effect (van der Grinten et al., 2010). A considerable higher *EC*_50_ value for trimethoprim (after 72 h of exposure) was also reported for *M. aeruginosa* (112 mg/L) (Halling-Sørensen, 2000) as well as for *Anabaena flos-aque* (253 mg/L) (Kolar et al., 2014) [obtained by toxicity assays according to OECD guidelines (OECD, 2011) for algal growth inhibition test]. Another study demonstrated that an *Anabaena* sp. and two *Nostoc* spp. strains were resistant to 25 μg of trimethoprim after exposure to antibiotic disks for 2–4 days on 0.8% agar medium (Prasanna et al., 2010). These studies can be hardly compared considering the differences in the experimental procedures, but they point out the reduced susceptibility of cyanobacteria to trimethoprim.

In our study we found that the four tested cyanobacteria genera were not susceptible to nalidixic acid (within the range of 0.0015–1.6 mg/L), but they exhibited distinct susceptibility degrees to norfloxacin, being *M. aeruginosa* particularly susceptible to this antibiotic (0.012 mg/L ≤ MIC ≤ 0.2 mg/L). The distinct response to these two antibiotics is not surprising considering that, among bacteria, nalidixic acid is a less potent quinolone than norfloxacine (Andersson and MacGowan, 2003). The bactericidal activity of quinolones occurs by the inhibition of the DNA gyrase and DNA topoisomerase IV, essential enzymes involved in DNA replication, and the mechanisms of quinolones resistance are due to modifications of these target enzymes or to changes in the antibiotic entry/efflux (Andersson and MacGowan, 2003; Jacoby, 2005). In Gram-negative bacteria the reduction of membrane permeability can be achieved by changes in the expression of porins (Jacoby, 2005). The cell wall from cyanobacteria has an overall structure similar to the Gram-negative cell wall, but their peptidoglycan layer is much thicker and their porins has a lower conductance (Hoiczyk and Hansel, 2000). The low cut-off of cyanobacterial porins is related with the protection against harmful agents such as toxins and antibiotics (Hoiczyk and Hansel, 2000). Considering these facts, we might hypothesize that the apparent reduced susceptibility to nalidixic acid of the four cyanobacteria genera we studied is due to their low permeability to this antibiotic. Besides, we also might consider that differences in cell wall permeability might also explain the higher susceptibility of *M. aeruginosa* (LMECYA 7) to norfloxacin, in comparison with other cyanobacteria, particularly the *P. agardhii* (LMECYA 260). It is known that peptidoglycan layer of cyanobacterial cell walls varies among genus/species, being considerable thicker in species from the Oscillatoriales order, to which *P. agardhii* belongs. Two previous studies have also reported that a strain of *Anabaena* sp. (Lorenz and Krumbein, 1984; Prasanna et al., 2010) and a strain of *Synechochoccus* were resistant to 30 μg/disk of nalidixic acid. However, the same authors also described a susceptible phenotype to the same dose of this antibiotic for another strain of *Anabaena* sp. and two strains of *Nostoc* sp. (Prasanna et al., 2010), as well as for a strain of *Pseudoanabaena* sp. (Lorenz and Krumbein, 1984). As for the other antibiotics, a definitive conclusion concerning the effects of quinololes on cyanobacteria is not possible to achieve with the available data.

Considering the cyanobacteria strains we conclude that *M. aeruginosa* (LMECYA 7) was the less susceptible to amoxicillin, but the most susceptible to norfloxacin. *A. gracile* (LMECYA 40) and *C. bergii* (LMECYA 246) were particularly susceptible to β-lactams. *P. agardhii* (LMECYA 260) were most susceptible to amoxicillin, but exhibited moderate to high MICs for other antibiotics. It is tempting to associate these differences with the cyanobacterial order since *M. aeruginosa* belongs to Chroococcales, *A. gracile* and *C. bergii* belong to Nostocales, and *P. agardhii* to Oscilatorialles orders. In fact, the consistency observed with the results from the nine tested *M. aeruginosa* strains (that were isolated from distinct freshwater reservoirs), supports the hypothesis that cyanobacteria specie might be related with specific antibiotic susceptibility patterns. On the other hand, the observation that all tested cyanobacteria were not susceptible to trimethoprim and nalidixic acid (from 0.0015 to 1.6 mg/L) supports the hypothesis that cyanobacteria, independently of the specie, might share common responses to antibiotics. However, further studies using a higher number of strains for the other species will be required in order to identify putative antibiotic susceptibility patterns related with cyanobacteria specie, genus or orders.

In conclusion, the reduced susceptibility of cyanobacteria to some antibiotics suggests that they may be naturally (constitutively) non-susceptible to these compounds or even that they might acquire antibiotic non-susceptibility due to environmental selection pressure by antibiotic exposure or to the transference of antibiotic resistance genes from bacteria. Considering that the available information on this issue is very dispersed and difficult to compare studies including species from a diversity of water bodies will be fundamental to map the resistance phenotypes/genotypes of cyanobacterial isolates from freshwater environments. Another fundamental aspect will be the establishment of standard procedures and breakpoints in order to define the MIC values to the different classes of antibiotics for cyanobacteria.

The knowledge on the role of cyanobacteria on the water resistome will help to understand how the aquatic ecosystems react to antibiotic pollution and to define preventive/remedial measures concerning the dissemination of antibiotic resistance in the environment.

## Author contributions

ED performed all the experiments with the help of MO, DJ, EF, and VM. ED, and MC wrote the manuscript. All the authors contributed to the design of the experiments, to the interpretation of data, to the revision of the manuscript and approved the version to be published.

### Conflict of interest statement

The authors declare that the research was conducted in the absence of any commercial or financial relationships that could be construed as a potential conflict of interest.

## Supplementary material

Supplementary Figure 1 **Conditions of antibiotic susceptibility test in cyanobacteria strains performed in 96-well microplates, with nine antibiotics (0.0015–1.6 mg/L)**.

Supplementary Figure 2 **Photographs of 96-well microplates containing the four cyanobacterial strains after 13 days of exposure to different classes of antibiotics within the concentration range of 0.0015–1.6 mg/L**. For each case, the green color denotes cyanobacterial growth. The MIC value corresponds to the well plate where the cell growth was totally inhibited (MIC values are listed in Table 1). The cyanobacterial strains were *M. aeruginosa* (LMECYA 7), *A. gracile* (LMECYA 40), *C. berghii* (LMECYA 246), and *P. agardhii* (LMECYA 260). Two inocula per strain were tested [5 × 10^5^cells/mL (inoculum 1) and 2 × 10^6^cells/mL (inoculum 2)], as follows: A, LMECYA 7/Inoculum 1; B, LMECYA 7/Inoculum 2; C, LMECYA 40/Inoculum 1; D, LMECYA 40/Inoculum 2; E, LMECYA 246/Inoculum 1; F, LMECYA 246/Inoculum 2; G, LMECYA 260/Inoculum 1; H, LMECYA 260/Inoculum 2. Plate (a) antibiotics that inhibited the cell growth at the lowest concentration; Plate (b) antibiotics that inhibited the cell growth at middle/high concentration; Plate (c) antibiotics that did not inhibited the cell growth within the tested concentrations.

Supplementary Table 1 **Minimun inhibitory concentrations (MIC, mg/L) of the tested antibiotics in standard *E. coli* and *S. aureus* ATCC strains, after being maintained in cyanobacterial culture conditions for 1, 7, and 14 days**.
