# Supplementary figures and images for: Assessing the antibiotic susceptibility of freshwater Cyanobacteria spp
Source: Front Microbiol. 2015 Aug 11;6:799. doi: 10.3389/fmicb.2015.00799 (PMC4531292; doi:10.3389/fmicb.2015.00799)

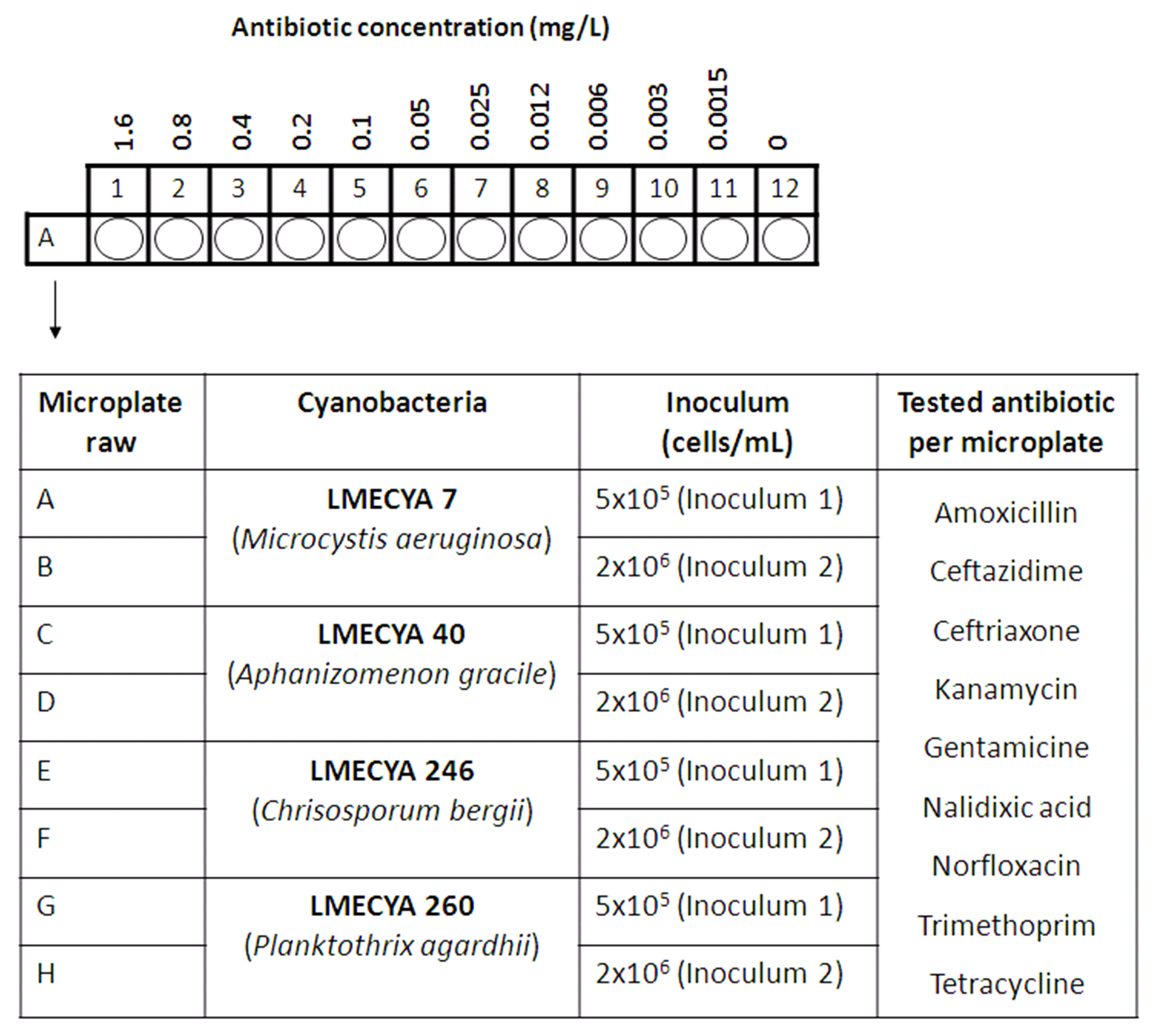

Supplement: Supplementary Figure 1 — Conditions of antibiotic susceptibility test in cyanobacteria strains performed in 96-well microplates, with nine antibiotics (0.0015–1.6 mg/L). [file Image1.TIF]

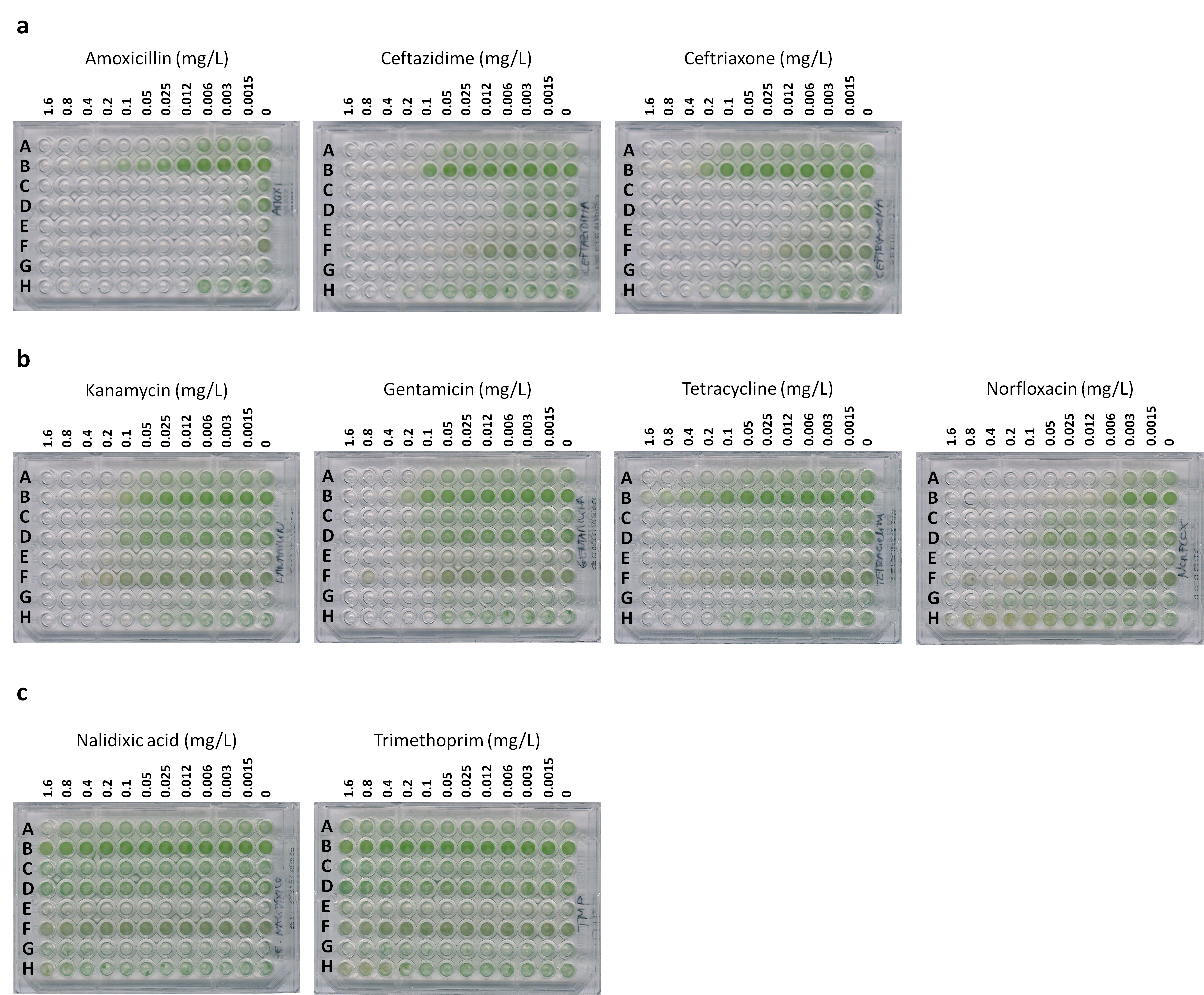

Supplement: Supplementary Figure 2 — Photographs of 96-well microplates containing the four cyanobacterial strains after 13 days of exposure to different classes of antibiotics within the concentration range of 0.0015–1.6 mg/L. For each case, the green color denotes cyanobacterial growth. The MIC value corresponds to the well plate where the cell growth was totally inhibited (MIC values are listed in Table 1). The cyanobacterial strains were M. aeruginosa (LMECYA 7), A. gracile (LMECYA 40), C. berghii (LMECYA 246), and P. agardhii (LMECYA 260). Two inocula per strain were tested [5 × 10^5^cells/mL (inoculum 1) and 2 × 10^6^cells/mL (inoculum 2)], as follows: A, LMECYA 7/Inoculum 1; B, LMECYA 7/Inoculum 2; C, LMECYA 40/Inoculum 1; D, LMECYA 40/Inoculum 2; E, LMECYA 246/Inoculum 1; F, LMECYA 246/Inoculum 2; G, LMECYA 260/Inoculum 1; H, LMECYA 260/Inoculum 2. Plate (a) antibiotics that inhibited the cell growth at the lowest concentration; Plate (b) antibiotics that inhibited the cell growth at middle/high concentration; Plate (c) antibiotics that did not inhibited the cell growth within the tested concentrations. [file Image2.TIF]
