# Supplementary material for: Assessing the antibiotic susceptibility of freshwater Cyanobacteria spp
Source: Front Microbiol. 2015 Aug 11;6:799. doi: 10.3389/fmicb.2015.00799 (PMC4531292; doi:10.3389/fmicb.2015.00799)
Supplement: Supplementary Table 1 — Minimun inhibitory concentrations (MIC, mg/L) of the tested antibiotics in standard E. coli and S. aureus ATCC strains, after being maintained in cyanobacterial culture conditions for 1, 7, and 14 days. [file Table1.DOCX]

**Suplement data Table I**- Minimun inhibitory concentrations (MIC, mg/L) of the tested antibiotics in standard *E. coli* and *S. aureus* ATCC strains, after being maintained in cyanobacterial culture conditions for 1, 7 and 14 days.

| **Antibiotics** | ***E. coli* (ATCC 25922)** | | | | | ***S. aureus* (ATCC 29213)** | | | | |
| --- | --- | --- | --- | --- | --- | --- | --- | --- | --- | --- |
|  | **Standard^a^** | 0h | 24h | 7d | 14d | **Standard^a^** | 0h | 24h | 7d | 14d |
| Amoxicillin | **2-8^b^** | 4 | 8 | 8 | 16 | **0.5-2^b^** | 1 | 4 | 4 | >4 |
| Ceftazidime | **0.06-0.5** | 0.25 | 0.25 | 0.25 | 1 | **4-16** | 8 | 8 | 16 | 16 |
| Ceftriaxone | **0.03-0.12** | 0.125 | 0.25 | 0.125 | 0.25 | **1-8** | 2 | 2 | 4 | 4 |
| Kanamycin | **1-4** | 4 | 4 | 8 | 4 | **1-4** | 4 | 4 | 4 | 8 |
| Gentamicin | **0.25-1** | 1 | 2 | 1 | 1 | **0.12-1** | 0.5 | 1 | 1 | >2 |
| Tetracycline | **0.5-2** | 1 | 2 | >4 | >4 | **0.12-1** | 0.25 | 1 | >2 | 2 |
| Trimethoprim | **0.5-2** | 0.5 | 1 | 1 | 1 | **1-4** | 1 | 2 | 1 | 4 |
| Nalidixic acid | **1-4** | 2 | 4 | 4 | 2 | **16-64^c^** | >32 | >32 | >32 | >32 |
| Norfloxacin | **0.03-0.12** | 0.06 | 0.06 | 0.25 | 0.125 | **0.5-2** | 2 | 2 | 2 | 2 |

^a^From ISO 20776-1:2006 Guidelines; ^b^Ampicilin-based ISO 20776-1:2006 Guidelines; ^c^Quality control range assigned by the Danish Veterinary Laboratory.
